# The Antibacterial Effect of Ethanol Extract of Polish Propolis on Mutans Streptococci and Lactobacilli Isolated from Saliva

**DOI:** 10.1155/2013/681891

**Published:** 2013-03-27

**Authors:** Arkadiusz Dziedzic, Robert Kubina, Robert D. Wojtyczka, Agata Kabała-Dzik, Marta Tanasiewicz, Tadeusz Morawiec

**Affiliations:** ^1^Department of Conservative Dentistry with Endodontics, Division of Medicine and Dentistry, Medical University of Silesia, Pl. Akademicki 17, 41-902 Bytom, Poland; ^2^Department of Pathology, School of Pharmacy and Laboratory Medicine, Medical University of Silesia, ul. Ostrogorska 30, 41-200 Sosnowiec, Poland; ^3^Department and Institute of Microbiology and Virology, School of Pharmacy and Laboratory Medicine, Medical University of Silesia, ul. Jagiellońska 4, 41-200 Sosnowiec, Poland; ^4^Department of Oral Surgery, Division of Medicine and Dentistry, Medical University of Silesia, Pl. Akademicki 17, 41-902 Bytom, Poland

## Abstract

Dental caries occurrence is caused by the colonization of oral microorganisms and accumulation of extracellular polysaccharides synthesized by *Streptococcus mutans* with the synergistic influence of *Lactobacillus* spp. bacteria. The aim of this study was to determine *ex vivo* the antibacterial properties of ethanol extract of propolis (EEP), collected in Poland, against the main cariogenic bacteria: salivary mutans streptococci and lactobacilli. The isolation of mutans streptococci group bacteria (MS) and *Lactobacillus* spp. (LB) from stimulated saliva was performed by in-office CRT bacteria dip slide test. The broth diffusion method and AlamarBlue assay were used to evaluate the antimicrobial activity of EEP, with the estimation of its minimum inhibitory concentration (MIC) and minimum bactericidal concentration (MBC). The biochemical composition of propolis components was assessed. The mean MIC and MBC values of EEP, in concentrations ranging from 25 mg/mL to 0.025 mg/mL, for the MS and LB were found to be 1.10 mg/mL *versus* 0.7 mg/mL and 9.01 mg/mL *versus* 5.91 mg/mL, respectively. The exposure to an extract of Polish propolis affected mutans streptococci and *Lactobacillus* spp. viability, exhibiting an antibacterial efficacy on mutans streptococci group bacteria and lactobacilli saliva residents, while lactobacilli were more susceptible to EEP. Antibacterial measures containing propolis could be the local agents acting against cariogenic bacteria.

## 1. Introduction

Dental caries, the most prevalent disease affecting humans, continues to be a common and global public health problem, in spite of confirmed decline in some parts of the world. Caries etiology, with the main focus on oral microbiology and cariogenic bacteria, is central to understanding the potential opportunities for and likely impact of new antimicrobial agents to reduce the caries burden worldwide. Prevention and control of dental caries activity is not restricted to a single technique but includes regular check-ups, routine use of fluoride-containing toothpastes, decreased sugar intake, topical fluorides application, and at-home rinsing with anti-plaque and antibacterial solutions.

Over the last several years, a worldwide trend has been observed in the use of natural products, due to their proven-by-evidence pharmacological effect on oral cavity environment in terms of efficient caries prevention. This tendency may be also applied to “over-the-counter” therapeutic and prophylactic products which are recommended for caries control and oral health maintenance. Propolis, a natural substance produced by honeybees, which has been widely consumed in the folk medicine since ancient times, seems to be a promising ingredient of topical formulations due to its multidirectional biological properties [1]. Apart from antibacterial activity, various studies have demonstrated that propolis has other beneficial properties, such as antioxidative, antifungal, antiviral, and anti-inflammatory ones [2, 3]. Additionally, antiproliferative action in human tumor cell lines has been observed [4–7].

Ethanol extract of propolis (EEP), an effective antimicrobial and anti-inflammatory agent, has been used commercially on the market as a component of toothpaste, mouth rinses, lozenges, and so forth. It is confirmed [8–11] that EEP demonstrates antimicrobial activity against Gram-positive cocci of *Streptococcus mutans, *a facultative anaerobic bacterium commonly found in human oral cavity (saliva and dental plaque) and a main contributor to tooth decay caused by biofilm formation. However, in medicine and dentistry, it still remains an underestimated compound. Relatively few studies were aimed at the influence of EEP on the growth of *Lactobacillus *spp. bacteria [12, 13], a second significant contributor to dental caries progression, acting in second stage of tooth decay development as a coexisting factor. Therefore, further investigations are needed to validate a dose required to eliminate cariogenic microorganisms within the oral cavity, avoiding local or systemic adverse reactions at the same time.

Propolis, a semisolid mixture of organic resin and wax, produced by honeybees (*Apis mellifera*), is used by bees to seal their honeycombs and to protect the entrance against intruders. It is assumed that the chemical composition of propolis comprises approximately 50% of resin and vegetable balm, 30% of wax, 10% of essential and aromatic oils, 5% of pollens, and 5% of other trace substances, including organic debris, depending on the place and time of collection [14, 15]. The constituents of propolis vary widely, depending on the climate, season, location, or year, and its chemical composition is not stable [14, 16].

Various aromatic compounds, mainly flavonoids and phenolics, seem to be the pharmacologically active constituents in propolis [17–19], which are well-known plant compounds that have unique and multidirectional antioxidant, antibacterial, antifungal, anti-inflammatory, and immunomodulative properties [20–22]. As an anti-inflammatory agent, propolis stimulates the immune system by promoting phagocytic activity and cellular immunity [23, 24] and improves the healing effects on epithelial tissues [14]. Additionally, propolis contains nonspecific elements, such as iron and zinc that are important for collagen synthesis [25]. Since some of the constituents composing synthetic cariostatic measures may cause adverse effects, there is an increased need to screen for new antimicrobial agents, which may act against plaque formation and against cariogenic bacteria. The bacteriostatic, bactericidal, and antiadherent activities of propolis on microorganisms connected with dental caries suggest its significant influence on dental caries control within the oral cavity environment [12, 13, 26]. The available extensive studies are mainly concerned with the mainly well-known “red” and “green” propolis, and the biochemical assays of Polish propolis compound are not yet common [27], in terms of its anticariogenic activity and biological effect on oral microorganisms. Moreover, the phenolic and flavonoid profile of propolis from the Southeast of Poland has not been thoroughly described yet.

Selected group of oral streptococci is closely related to *Streptococcus mutans* and is referred to as the “mutans group” or the “mutans streptococci” [28]. Only two cariogenic species from the “mutans group”, *Streptococcus mutans* and *Streptococcus sobrinus*, are found commonly in human oral environment. The others were isolated in various animals (rats, macaque monkeys) [29]. Distinguishing of *S. mutans* and *S. sobrinus* in saliva by appropriate laboratory tests, to identify to the single species level, is rarely practicable, particularly in large-scale epidemiological studies. Most scientific sources regarding the relationship between oral streptococci and caries have considered the two species together as the mutans streptococci (MS). Because of a greater prevalence, most of the isolates are described as *S. mutans*, and sometimes the single name *S. mutans* is used erroneously, even though there was no investigation towards *S. sobrinus* presence and isolation. In the past, most papers were referred to *S. mutans* because *S. sobrinus* was not officially recognized.

The study aimed at investigating *in vitro* the antimicrobial activity of ethanol extract of Polish propolis against two main cariogenic oral pathogens with its effects on the mutans streptococci and lactobacilli growth. The solution prepared with propolis extract was also analyzed for its biochemical content and its qualitative antibacterial potential. The determination of optimal concentration of the Polish propolis against the clinical isolates from the saliva can support the development of the oral hygiene products, such as gargling solution and toothpastes.

## 2. Material and Methods

### 2.1. Preparation of Ethanol Extract of Propolis

Propolis samples were produced by honeybees (*Apis mellifera*) from the apiary in Kamianna (Nowy Sacz Voivodeship, south of Poland) which constituted the material for the research. The tree population in the area consists primarily of the black poplar (*Populus nigra*), birch (*Betula alba*), alder (*Alnus glutinosa*), beech (*Fagus sylvatica*), and horsechestnut (*Aesculus hippocastanum*). Hand-collected propolis was kept desiccated and in the dark before its processing. The samples were ground mechanically and bottled in 10 g portions. The portions of 10 g were put into flasks, and 100 g of 70% ethanol (w/v, POCH S.A., Poland) was added. Propolis was subjected to 14 days of extraction in order to obtain ethanol extract of propolis (EEP). The flask was placed in laboratory shaker in a dark, closed bottle for the time of two weeks in room temperature. After that time, the extract was cooled in 4°C for 24 hours in order to precipitate all insoluble substances. Rough particles were removed from the propolis extract by filter and filtered through filter paper (Whatman no. 4, UK). The filtrate obtained that way was evaporated, using rotary vacuum evaporator (Rotavapor R-215, BUCHI Labortechnik AG, Switzerland), in 40°C. This way, a viscous substance having brown colour was obtained, which was later dissolved in ethanol in order to receive 100 mg/mL of the working concentration.

### 2.2. Cariogenic Bacteria Isolation

The isolation and estimation of bacterial growth of mutans streptococci (MS) and *Lactobacillus *spp. (LB) was performed by means of semiquantitative method, using a dip slide, commercial screening CRT bacteria medium (Ivoclar-Vivadent, Liechtenstein). Seventeen adult subjects, aged 27–54, who were involved in this study, underwent a routine dental treatment at Department of Conservative Dentistry with Endodontics and at Academic Center of Dentistry and Specialist Medicine, Medical University of Silesia, Bytom, Poland. Inclusion criteria for the research group were based on medical and dental history, socioeconomic status, analysis of clinical documentation, assessment of oral hygiene (plaque indexes), and dental charting profile (DMFT index >3). The exclusions criteria comprised current treatment with antibiotics, the use of antibacterial mouth rinse or antiseptic lozenges, acute oral infections, and the severely decreased saliva flow (dry mouth syndrome). None of the participants refused to take part in the study, and informed consent for all patients was obtained, including adequate information to meet necessary requirements for the study.

CRT bacteria chair side test was used to isolate the mutans streptococci and lactobacilli from saliva, by means of selective culture media. Findings of 100.000 CFU or more of MS and LB per 1 mL of saliva indicate high caries risk. The indication of CRT bacteria includes *in vitro* diagnostics of the main cariogenic microbiota in saliva. The bright green agar surface is designed for the determination of LB count in saliva and the blue agar surface for determination of MS count in saliva, or plaque (MSB Agar). Diagnostic kits and selective media, designed for use in the dental clinic for isolating caries-related microorganisms, are based on selective media, and they are measuring total mutans streptococci count, not just *Streptococcus mutans* species. They may contain the specific ingredients, including antibiotics (bacitracin), which suppress the growth of most species but allows *S. mutans* and *S. sobrinus* to grow. 

The investigator collected stimulated saliva from each individual, according to the manufacturer's specification. Saliva samples were stimulated with paraffin-based sticks (1 minute) and collected into sterile flasks (2 mL on the average). The tablets of NaHCO_3_ were placed at the bottom of the vials. All samples were collected within a single working day (9 hours, from 9.00 till 18.00) by the same examiner, using the same technique and procedure. The vials were then immediately seeded in the laboratory based in Department and Institute of Microbiology and Virology (Sosnowiec, Poland), Medical University of Silesia. The test vials were placed upright in the incubator and incubated at 37°C/99°F for 48 hours. After removal of the vial from the incubator, the density of the MS and LB colonies was compared with the corresponding evaluation pictures in the enclosed model chart. The values of <10^5^ and >10^5^ were recorded for the low and high CFU ranking, based on the scale provided in the CRT kit (Ivoclar-Vivadent, Liechtenstein). Each sample was examined by the same viewer. Bacteria strains, mutans streptococci and* Lactobacillus *spp. isolated from clinical specimens, were subjected to further inoculation.

### 2.3. Determination of Minimum Inhibitory Concentration (MIC) and Minimum Bactericidal Concentration (MBC)

In order to establish the minimum inhibitory concentration (MIC) and the minimum bactericidal concentration (MBC) of isolated mutans streptococci and lactobacilli, the broth dilution method was used, as recommended by the Clinical and Laboratory Standards Institute [30, 31]. The minimum inhibitory concentration (MIC) was determined as the lowest concentration of the propolis extract, which inhibited the growth of the tested microorganisms. The MBC was defined as the lowest concentration of antimicrobial agents required to eradicate a particular bacterium. The MIC value has been determined by incubation of the isolated strains in 96-well microplates for 24 hours, at the temperature of 37°C. The bacterial inoculum has been prepared in 0.9% sodium chloride (POCH S.A., Poland) from fresh cultures. MIC value was estimated by visual and spectroscopic method by absorbance measurement at 600 nm (OD600—opticaldensity reading at 600 nm). 

The turbidity of the suspension has been adjusted to the McFarland 0.5 turbidity standard (Densi-La-Meter II, Erba Lachema, Brno, Czech Republic), and the inoculum has been diluted (1 : 100) in sterile medium. Serial two-fold dilutions of EEP have been prepared in Mueller-Hinton Broth II (MHB, Oxoid Ltd, Basingstone, Hempshire, UK), and ethanol was used as controls. The microorganisms have been exposed to serial dilutions of ethanol extract of propolis, within the range of 25 mg/mL to 0.025 mg/mL. The maximum concentration 25 mg/mL was then excluded from the experimental protocol. The inoculum size has been verified by plating serial dilutions of the inoculum and performing colony counts. The values of minimum and maximum MIC have been determined, as well as MIC_50_ and MIC_90_.

In order to determine the MBC value, from the MIC concentration and two higher concentrations, 10 *μ*L of the medium have been collected and transferred to 10 mL of sterile physiological saline. Subsequently, a series of three dilutions have been performed, in the proportion of 1 : 10. From each dilution, 100 *μ*L have been collected and disseminated on Mueller-Hinton medium, with the addition of 5% of ram blood, using surface culture method. The entire stuff incubated for 24 h in the temperature of 37°C. Petri dishes on which the number of colonies grown has been less than 300 have been considered for assessment. MBC has been considered achieved when a 99.9% reduction of the number of colonies has been achieved, in comparison with control. The control test has been carried out in an analogous way, with the addition of EEP.

### 2.4. AlamarBlue Susceptibility Colorimetric Assay

Planktonic susceptibility testing of mutans streptococci has been performed by the reference broth microdilution assay, using round-bottom, polystyrene, nontissue culture-treated microtiter microplates, and cation-adjusted Mueller-Hinton II Broth, as an additional method for MIC/MBC validation. After 24 hours of incubation, from each dilution of a volume 100 *μ*L have been collected, transferred on the microplates, and 5 *μ*L AlamarBlue (Invitrogen, US) was added to the wells (105 *μ*L total volume = 100 *μ*L of Mueller-Hinton II Broth, EEP, bacteria + 5 *μ*L AlamarBlue). The microplates were shaken gently and incubated for 2 h at 37°C. The plates were gently shaken again, and absorbance at 570 nm and 600 nm was obtained in a Multiskan EX microplate reader (Thermo Scientific, USA). 

For experiments with multiple time points, the microplates were kept in an incubator at 37°C between absorbance readings. Controls included media alone, media plus AlamarBlue (AB), media plus AlamarBlue plus propolis dilution, and cells plus media plus AlamarBlue. The percentage reduction of AlamarBlue (%AB) was calculated using the manufacturer's formula, with replacement of the negative control, which contains only media plus AlamarBlue, with a more robust negative control, media plus AlamarBlue plus a drug concentration equal for each experimental well:

(1) $$\begin{array}{c} \frac{{\left(\varepsilon_{\text{OX}}\right)}_{\lambda_{2}}A_{\lambda_{1}}-{\left(\varepsilon_{\text{OX}}\right)}_{\lambda_{1}}A_{\lambda_{2}}}{{\left(\varepsilon_{\text{OX}}\right)}_{\lambda_{2}}A_{\lambda_{1}}^{o}-{\left(\varepsilon_{\text{OX}}\right)}_{\lambda_{1}}A_{\lambda_{2}}^{o}}\times 100. \end{array}$$

In the formula, *ε*_*λ*_1__ and *ε*_*λ*_2__ are constants representing the molar extinction coefficient of AB at 570 and 600 nm, respectively, in the oxidized (*ε*_OX_) form. *A*_*λ*_1__ and *A*_*λ*_2__ represent absorbance of test wells at 570 and 600 nm, respectively. *A*_*λ*_1__^*o*^ and *A*_*λ*_2__^*o*^ represent absorbance of positive control wells at 570 and 600 nm, respectively. The values of %AB reduction were corrected for background values of negative controls containing medium without cells.

Assays were performed at least twice, and the average percentage reduction was used to determine the MIC. AlamarBlue MIC was defined as the lowest ethanol extract of propolis concentration resulting in ≤50% reduction of AB (average of two experiments) and a purple/blue well 120 minutes after the addition of AB.

### 2.5. Biochemical Assay of Polyphenolic and Flavonoid Constituents

The total polyphenolic content in propolis was determined using the Folin-Ciocalteu colorimetric method (Spekol 11, Carl Zeiss, Jena, Germany). A reference mixture of pinocembrin and galangin (2 : 1, w/w, Sigma-Aldrich, USA) was prepared, after it was further diluted into a series of appropriate concentrations (from 0.021 to 0.335 mg/mL) that were used for the calibration curve. One mL of the test solution was transferred to a 50 mL volumetric flask, containing 15 mL distilled water. Then, 4 mL of the Folin-Ciocalteu reagent (Merck Millipore, USA) and 6 mL of a 20% sodium carbonate solution were added. The volume was adjusted to 50 mL with distilled water. The colorimetric absorbance was measured at 760 nm after 2 h. 

The total flavonoid content was determined by quantification of the flavones/flavonols and flavanone/dihydroflavonols [32]. Stock standard solutions of galangin (0.04 mg/mL) for flavones/flavonols assay and pinocembrin (1 mg/mL) for the flavanone/dihydroflavonols assay were prepared in order to construct the calibration curves. The series of five dilutions in the range of 0.005–0.04 mg/mL for galangin and 0.1–0.8 mg/mL for pinocembrin were also prepared.

An aliquot of 1 mL of the test solution and 0.5 mL of 5% aluminum chloride (POCH S.A., Poland) in methanol (POCH S.A., Poland) were mixed in a flask containing 10 mL of methanol. The volume was adjusted to 25 mL with methanol; after 30 minutes the absorbance was measured at 425 nm against the blank in order to quantify flavones/flavonols. An aliquot of the initial extract of propolis (1 mL) and 2 mL of 2,4-Dinitrophenylhydrazine solution (POCH S.A., Poland, 1 g of DNP was mixed with 2 mL of 96% sulphuric acid and diluted to 100 mL with methanol) were heated at 50°C for 50 min. After cooling down to room temperature, the solution was diluted to 10 mL with 10% potassium hydroxide methanolic solution (POCH S.A., Poland). Half of the one mL of the solution was transferred into a volumetric flask, and the volume was adjusted to 25 mL with methanol. The absorbance was measured at 486 nm against the blank, in order to quantify flavanones and dihydroflavonols.

Blank solutions were prepared by replacing the sample with an equivalent aliquot of methanol that was carried out through all the steps of the applied procedure. The results obtained are presented as percentage ± standard deviation (± SD). The content of other phenols was assessed by subtracting the content of total flavonoids from the content of total polyphenols.

## 3. Results

**Figure 1:**
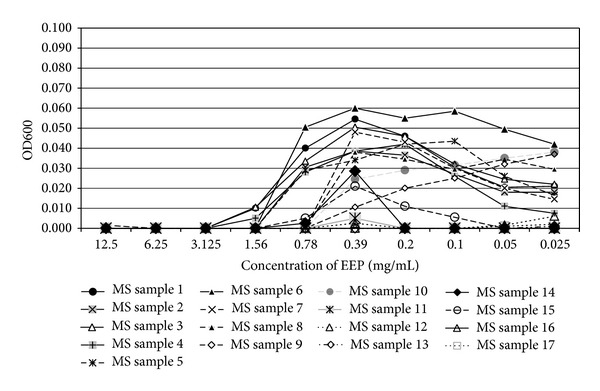
The influence of different concentrations of EEP on absorbance changes for MS (*n* = 17) during 1 hour of EEP activity (OD600—optical density reading at 600 nm).

**Figure 2:**
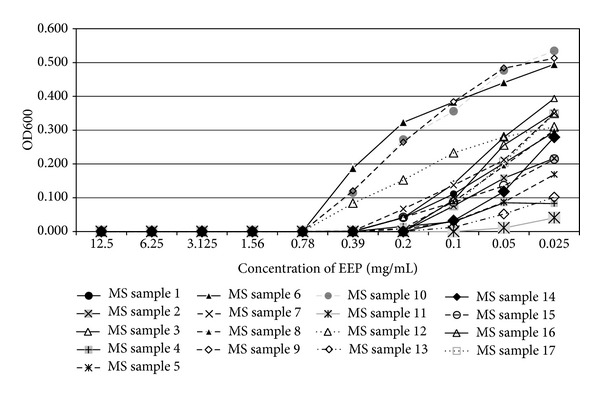
The influence of different concentrations of EEP on absorbance changes for MS (*n* = 17) during 4 hours of EEP activity (OD600—optical density reading at 600 nm).

**Figure 3:**
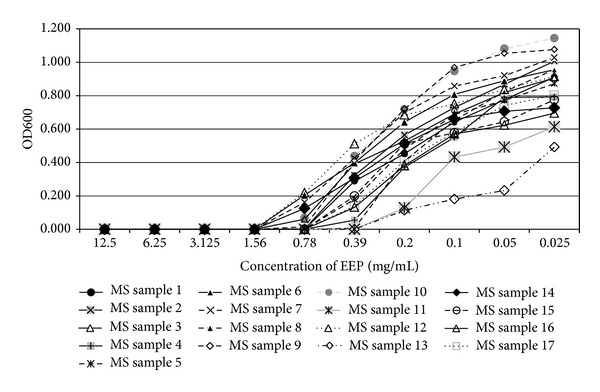
The influence of different concentrations of EEP on absorbance changes for MS (*n* = 17) during 24 hours of EEP activity (OD600—optical density reading at 600 nm).

**Figure 4:**
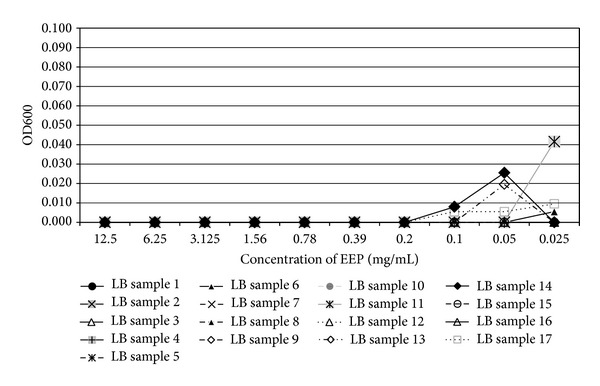
The influence of different concentrations of EEP on absorbance changes for *Lactobacillus* spp. (*n* = 17) during 1 hour of EEP activity (OD600—optical density reading at 600 nm).

**Figure 5:**
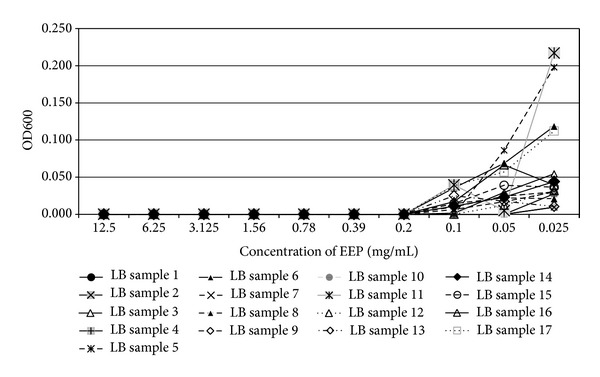
The influence of different concentrations of EEP on absorbance changes for *Lactobacillus* spp. (*n* = 17) during 4 hours of EEP activity (OD600—optical density reading at 600 nm).

**Figure 6:**
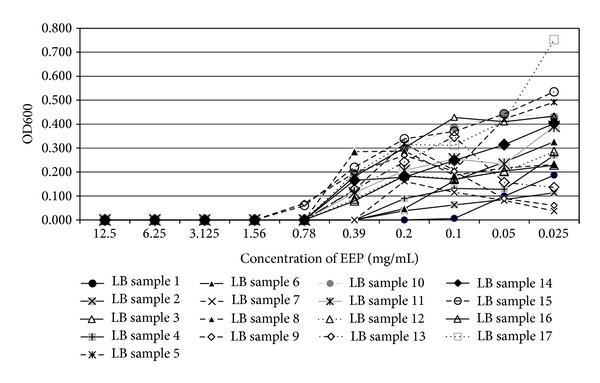
The influence of different concentrations of EEP on absorbance changes for *Lactobacillus* spp. (*n* = 17) during 24 hours of EEP activity (OD600—optical density reading at 600 nm).

In the present study, the examined extract of Polish propolis with ethanol demonstrated *in vitro *antimicrobial activity against main cariogenic bacteria, that is, mutans streptococci group and *Lactobacillus *spp. (Tables 1, 2, 3, and 4). The exposure of microorganisms to propolis (0.025–24 mg/mL) for 24 h affected bacteria viability, as was measured and confirmed quantitatively by the AlamarBlue assay. Figures 1, 2, and 3 demonstrate the influence of different concentrations of EEP on absorbance changes for mutans streptococci during 1–4–24 hour(s) of EEP activity. Figures 4, 5, and 6 represent the influence of different concentrations of EEP on absorbance changes for *Lactobacillus *spp. during 1–4–24 hour(s) of EEP activity.

**Figure 7:**
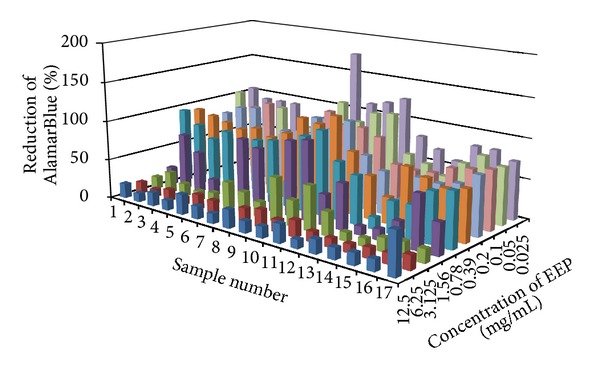
Percentage reduction of AlamarBlue at 120 minutes for mutans streptococci (*n* = 17) treated for 24 hours with ethanol extract of Polish propolis at the concentrations from 12.5 to 0.025 mg/mL.

The mean MIC value of EEP was found to be 1.10 ± 0.45 mg/mL for mutans streptococci and 0.7 ± 0.29 mg/mL for lactobacilli (Table 2), while the mean MBC value of EEP was found to be 9.01 ± 3.85 mg/mL for mutans streptococci and 5.91 ± 3.62 mg/mL for lactobacilli (Table 4). The MBC for each sample was a multiple value of its mean MIC. AlamarBlue assay quantitative results for MS group were in correlation with mean MIC values and mean AlamarBlue MIC values for MS group reached, in a vast majority of cases, a double mean MIC value (2x).  Figure 7 presents the percentage reduction of AlamarBlue at 120 minutes for mutans streptococci treated for 24 hours with ethanol extract of propolis at the concentrations from 12.5 to 0.024 mg/mL. AlamarBlue reduction was increasing with incubation time and with lower EEP concentration. Changes of medium colour associated with AB reduction from blue (oxidized) to pink (reduced) in duplicate wells.

**Figure 8:**
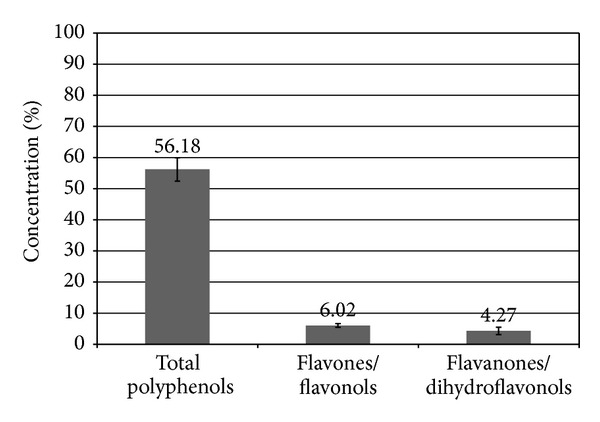
Total polyphenol and flavonoid content in investigated propolis sample from Poland. Concentrations (% ± standard deviation) of total polyphenols, flavones/flavonols, and flavanones/dihydroflavonols.

Based on biochemical analysis, EEP phenolic composition generally fits well with that of propolis from different countries, with other organic ingredients. The total polyphenols content was established as 56.18 ± 7.53%, while the total flavones/flavonols amount and flavanone/dihydroflavonols content amounted to 6.02 ± 1.23% and 4.27 ± 2.36%, respectively (Figure 8).

## 4. Discussion

The available *in vitro* and *in vivo* studies have reported potential application of propolis in the control of dental caries, especially since it has already been incorporated into commercial domestic products for oral use [12, 33, 34]. The mechanism of antimicrobial action demonstrated by propolis, including cariogenic microorganisms, is controversial and not completely understood. The biological activity of propolis (EEP) may vary according to its composition and seems to be multidirectional [35], involving several mechanisms such as the disorganization of the cytoplasmatic membrane and the cell wall; partial bacteriolysis; formation of pseudomulticellular colonies; and inhibition of protein synthesis [36]. It is assumed that the synergistic effect of main components of propolis extracts like flavonoids (quercetin, galangin, pinocembrin) and caffeic acid and/or cinnamic acid, probably influence the microbial membrane or cell wall sites, resulting in functional and structural effects [37–40].

The significant variability of the chemical composition of propolis may be a limitation in terms of its quality control, comparability, and reproductive effect [41]. Therefore, a critical analysis of the data available on propolis is essential. Elbaz and Elsayad [13] compared the antimicrobial affect of Egyptian propolis *versus* propolis from New Zealand on *Streptococcus mutans *and* Lactobacillus spp.* in saliva and found that the commercially available propolis from New Zealand, hexane fraction, had the strongest antimicrobial action. 

The antimicrobial activity of propolis is widely supported by evidence [42]. Some authors found propolis samples to be active only against gram-positive bacteria and some fungi [15, 25]; however, others confirmed also its weak activity against gram-negative bacteria [8]. The experimental propolis solution investigated by Ozan et al. demonstrated significant effect on gram-positive strains as on gram-negative strains [14] and also showed sufficient effect on gram-negative strains and on *Candida* strains. This study is in accordance with Sforcin et al. [8], who verified that the growth of Gram-positive bacteria was inhibited by low propolis concentrations (0.4%), with the MIC ranging from 4.5% to 8.0%. Drago et al. [43] also observed that in low concentrations propolis reveals bacteriostatic rather than bactericidal activity. 

Our findings demonstrated the antibacterial effect of Polish propolis on planktonic mutans streptococci and lactobacilli collected from saliva. The susceptibility of microbiota, which belongs to Streptococcus mutans group and initiating dental caries by teeth demineralization, has been slightly lower than in the case of *Lactobacillus *spp. (1.10 mg/mL mean MIC SM > 0.7 mg/mL mean MIC LB), the microorganisms responsible for caries progression. Moreover, the minimum MIC value estimated for *Lactobacillus *spp. was significantly lower than found for* S. mutans* (0.2 mg/mL *versus *0.39 mg/mL). These concentrations can be used *in vivo *for the prevention of dental caries. The results were opposite to the data presented by other authors [12], who concluded that EEP had a more potent antimicrobial effect on *S. mutans* than on *Lactobacillus *spp. These results also reflect the fact that oral microorganisms susceptibility to EEP varies and depends on genetic profile of specific individual microflora present in saliva. The research investigating the effect of propolis on dental caries in rats [44, 45] confirmed that propolis revealed antimicrobial activity against *S. sobrinus*, *S. mutans*, and *S. cricetus* as well as inhibited both water-insoluble glucan synthesis and glucosyltransferase activity. The investigations revealed the significant influence of *Streptococcus mutans* counts, which are considered major factors in the progression of cariogenic process [46, 47]. On the other hand, a single clinical study revealed that propolis was no better than placebo in inhibiting dental plaque formation [48]. Based on *in vitro* studies, Kim et al. stated that Korean propolis at concentrations >35 *μ*g/mL has antimicrobial activity against 90% of the mutans streptococci strains (55 strains) isolated from Koreans [49]. Interestingly, the MIC_50_ and MIC_90_ values, representing Korean propolis concentration, were the same for *S. mutans *and *S. sobrinus* species (35 *μ*g·mL). These results suggest that susceptibility of two main mutans streptococci species to propolis is similar and comparable. 

The determination of MIC values depends on technical details that may vary significantly between laboratories and is linked to the bacteria inherent virulence and susceptibility. In our study, the MIC and MBC values were at a relatively low level. Overall, MICs for mutans streptococci determined visually and colorimetrically by AlamarBlue assay were highly correlated with those determined in traditional method. Microplate-based assay which uses AlamarBlue reagent for determination of cells (bacteria) growth seems to be a rapid, low-cost technology for antimicrobial drug screening, which does not require expensive instrumentation. Oxidation-reduction dyes have been used to obtain drug susceptibility measurements for bacteria [50]. Yajko et al. [51] reported as a result of tests with clinical isolates a good correlation between the proportion technique and a novel method with Alamar Blue, a proprietary, resazurin-based [52], and oxidation-reduction indicator which delivered colorimetric MICs for cells, including microbiota. 

In the study, investigating antibacterial effects of glass-ionomer cement containing ethanolic extract of propolis on *Streptococcus mutans* [1], the authors demonstrated that MIC values of Turkish propolis for *S. mutans* ATCC 25175 amounted to 25 *μ*g/mL. According to disk diffusion test results, the experimental GICs containing EEP exhibited inhibition zones, and the inhibition zone sizes were not dependent upon the concentration of propolis. According to Ophori et al. [53], the EEP at the concentrations of 4, 8, 16, and 32 *μ*g/mL showed strong antimicrobial activity against *S. mutans *with inhibition zones of 10 ± 4, 12 ± 4, 20 ± 2, and 24 ± 2 mm, respectively, with the use of several dilutions of EEP, ranging between 0.5 and 32 *μ*g/mL. Some authors [33] reported that the EEP MIC for *Streptococcus mutans* ranged from 80 to 40 mL (8.8 mg to 4.4 mg of propolis) and the MIC value of the extract of propolis without alcohol for *Streptococcus mutans *ranged from 40 to <10 mL (4.4 to <1.1 mg of propolis). They investigated two samples of Brazilian commercially available propolis: ApisFlora 11.0% ethanolic extract of propolis and Propomax 11.0% extract of propolis without alcohol. 

The association between oral microorganisms found in the saliva as nonadhering populations and as plaque, a microbial biofilm, and specific oral conditions such as dental caries and periodontal disease has been widely described [54]. Clinical studies demonstrated that propolis may prevent caries development. Koo et al. [55] stated that mouth rinse containing propolis showed significant reduction of dental plaque compared to the placebo and also significant inhibition of insoluble polysaccharide formation. Data from clinical studies have also demonstrated reductions in *Streptococcus mutans* collected from saliva in *ex vivo* conditions. They showed the influence of EEP on plaque index score and insoluble polysaccharide formation, responsible for dental plaque accumulation [14, 34, 56, 57]. These results indicate that propolis and/or its compounds are promising cariostatic agents. According to the study results presented by Malhotra et al., the laboratory manufactured propolis mouth rinse showed an effective antimicrobial action only against *Streptococcus mutans* [12]. The antimicrobial efficacy of propolis against *Streptococcus mutans* was similar to that of chlorhexidine and the combination of propolis with chlorhexidine.

Because of the fact that the planktonic forms of bacteria in saliva are less resistant than their forms in biofilms (dental plaque), the MIC value does not provide sufficient information concerning the efficacy of antimicrobial agents against infections involving biofilms. It needs to be emphasized that the variation in the chemical composition of propolis due to its geographical distribution is a significant drawback to its routine clinical use as a preventive measure against dental caries. Therefore, more relevant clinical studies are needed to establish quality control protocol for propolis-containing agents, used in accordance with its confirmed activity.

## 5. Conclusions

This study showed a positive inhibitory influence of ethanol extract of Polish propolis with respect to the oral microorganisms growth. The antibacterial effect of propolis seems to be a representation of the synergistic activity of polyphenolics and other organic ingredients. It can be concluded that local measures, for example, lozenges and mouthwashes containing propolis, would be promising agents for dental plaque and caries control, including cariostatic effect. Reduction in oral flora counts obtained by antibacterial efficacy of propolis-based measures may provide an alternative approach for the individuals with high risk of dental caries. Further studies should be performed on propolis biological aspects to establish how the presence of ethanol extract of propolis interferes with the other adjuvants and active anticariogenic substances. Also, additional research is needed to validate clinical results in terms of other bacterial environments (dental plaque).

## Figures and Tables

**Table 1:** Mean MIC values of mutans streptococci and lactobacilli for each sample.

Sample no. (mutans streptococci)	Mean MIC (mg/mL)	Mean AlamarBlue MIC for SM (mg/mL)	CRT bacteria (CFU)	Sample no. (*Lactobacillus* spp.)	Mean MIC (mg/mL)	CRT bacteria (CFU)
1	0.78	1.56	<10^5^	1	0.20	<10^5^
2	1.56	3.12	<10^5^	2	0.39	<10^5^
3	1.56	3.12	<10^5^	3	0.78	<10^5^
4	0.78	1.56	<10^5^	4	0.39	<10^5^
5	0.78	0.78	<10^5^	5	0.78	<10^5^
6	1.56	3.12	>10^5^	6	0.39	>10^5^
7	1.56	3.12	<10^5^	7	0.39	>10^5^
8	0.78	0.78	<10^5^	8	0.78	<10^5^
9	1.56	6.25	<10^5^	9	1.56	>10^5^
10	1.56	3.12	<10^5^	10	0.78	>10^5^
11	0.39	1.56	<10^5^	11	0.78	<10^5^
12	1.56	3.12	<10^5^	12	0.78	<10^5^
13	0.39	0.20	>10^5^	13	0.78	>10^5^
14	1.56	0.78	<10^5^	14	0.78	<10^5^
15	0.78	0.78	<10^5^	15	0.78	<10^5^
16	0.78	3.12	>10^5^	16	0.78	>10^5^
17	0.78	1.56	<10^5^	17	0.78	<10^5^

**Table 2:** MIC values of ethanol extract of propolis for MS (*n* = 17) and LB (*n* = 17) isolated from human saliva.

Cariogenic pathogens	Mean MIC ± SD (mg/mL)	Min MIC (mg/mL)	Max MIC (mg/mL)	MIC_50_ (mg/mL)	MIC_90_ (mg/mL)	% Susceptible bacteria*
Mutans streptococci	1.10 ± 0.45	0.39	1.56	0.78	1.56	100
*Lactobacillus* spp.	0.7 ± 0.29	0.20	1.56	0.78	0.78	100

Cariogenic pathogens	Mean AlamarBlue MIC ± SD (mg/mL)	Min AlamarBlue MIC (mg/mL)	Max AlamarBlue MIC (mg/mL)	AlamarBlue MIC_50_ (mg/mL)	AlamarBlue MIC_90_ (mg/mL)	% Susceptible bacteria*

Mutans streptococci	2.13 ± 1.53	0.16	6.25	1.56	3.12	100

MIC_50_, MIC_90_, and MIC_100_—minimal inhibitory concentration needed to inhibit the growth of 50, 90, and 100% of mutans streptococci and lactobacilli, respectively.

(*) 100% of tested bacteria were susceptible to range of EEP concentrations.

**Table 3:** Mean values of MBC for mutans streptococci and *Lactobacillus* spp. for each sample.

Sample no. (mutans streptococci)	Mean MBC (mg/mL)	Sample no. (*Lactobacillus* spp.)	Mean MBC (mg/mL)
1	6.25	1	0.39
2	12.5	2	12.5
3	12.5	3	12.5
4	3.13	4	1.56
5	6.25	5	3.13
6	12.5	6	12.5
7	12.5	7	1.56
8	3.13	8	3.13
9	12.5	9	6.25
10	12.5	10	6.25
11	6.25	11	6.25
12	12.5	12	6.25
13	3.13	13	3.13
14	6.25	14	6.25
15	12.5	15	6.25
16	12.5	16	6.25
17	6.25	17	6.25

**Table 4:** MBC values of ethanol extract of propolis for mutans streptococci (*n* = 17) and *Lactobacillus* spp. (*n* = 17) isolated from human saliva.

Cariogenic pathogens	Mean MBC ± SD (mg/mL)	Min MBC (mg/mL)	Max MBC (mg/mL)	MBC_50_ (mg/mL)	MBC_90_ (mg/mL)	% Susceptible*
MS	9.01 ± 3.85	3.13	12.5	12.5	12.5	100
LB	5.91 ± 3.62	0.39	12.5	6.25	12.5	100

MIC_50_, MIC_90_, and MIC_100_—minimal inhibitory concentration needed to inhibit the growth of 50, 90, and 100% of mutans streptococci and lactobacilli, respectively.

(*) 100% of tested bacteria were susceptible to range of EEP concentrations.
